# Seed preference is only weakly linked to seed-type-specific feeding performance in a songbird

**DOI:** 10.1242/bio.060353

**Published:** 2024-04-11

**Authors:** Tim Andries, Wendt Müller, Sam Van Wassenbergh

**Affiliations:** ^1^ Laboratory of Functional Morphology, Department of Biology, University of Antwerp, Universiteitsplein 1, 2610 Antwerpen, Belgium; ^2^ Behavioural Ecology and Ecophysiology Research Group, Department of Biology, University of Antwerp, Universiteitsplein 1, 2610 Antwerpen, Belgium

**Keywords:** Beak kinematics, Feeding performance, Feeding skills, Seed preference, Songbirds

## Abstract

The dehusking of seeds by granivorous songbirds is a complex process that requires fast, coordinated and sensory-feedback-controlled movements of beak and tongue. Hence, efficient seed handling requires a high degree of sensorimotoric skill and behavioural flexibility, since seeds vary considerably in size, shape and husk structure. To deal with this variability, individuals might specialise on specific seed types, which could result in greater seed handling efficiency of the preferred seed type, but lower efficiency for other seed types. To test this, we assessed seed preferences of canaries (*Serinus canaria*) through food choice experiments and related these to data of feeding performance, seed-handling skills and beak kinematics during feeding on small, spindle-shaped canary seeds and larger, spheroid-shaped hemp seeds. We found great variety in seed preferences among individuals: some had no clear preference, while others almost exclusively fed on hemp seeds, or even prioritized novel seed types (millet seed). Surprisingly, we only observed few and weak effects of seed preference on feeding efficiency. This suggests either that the ability to handle seeds efficiently can be readily applied across various seed types, or alternatively, that achieving high levels of seed-specific handling skills does not require extensive practice.

## Teaser

**Summary:** We studied how seed preference in canaries affects an individual's performance in handling various seed types. Surprisingly, despite considerable variability in preference, this only had weak influence on feeding performance.

## INTRODUCTION

Individuals within populations of the same species can differ in dietary preferences (Bolnick et al., 2002, 2011; Des Roches et al., 2018). Although the degree of this individual variation varies across taxa, this is generally considered to be a widespread phenomenon (Bolnick et al., 2003). Individual specialization in diet is generally expected to arise as a way to reduce intraspecific competition over food, which is typically a limited resource in nature (Futuyma and Moreno, 1988). The extent of this specialization is then related to the degree of spatio-temporal food predictability. Generalists are hypothesized to flexibly adjust to environmental stimuli and thrive in conditions with low food predictability. On the other hand, specialists are thought to rely more on engrained strategies to respond in a more integrated fashion to environmental conditions, and thus benefit more from high food predictability (Bolnick et al., 2003; Wakefield et al., 2015). In addition, dietary specialization is often found to be related to sex (males and females specialize in different food sources, e.g. Shine, 1989), age (individuals change their diet as they mature, e.g. Polis, 1984) or morphology (morphs of polymorphic species differ in dietary specialization, also known as resource polymorphism, e.g. Skulason and Smith, 1995), again to reduce competition.

Granivorous songbirds in particular have long been of great interest to study diet specialization. As seeds vary greatly in size, shape and structure and songbirds need to dehusk them prior to ingestion, granivory is an advanced feeding strategy that requires fast, complex and well-coordinated movements of beak and tongue (Mielke and Van Wassenbergh, 2022; Nuijens and Zweers, 1997; van der Meij and Bout, 2006). It is hence a mechanically challenging endeavour, which provides opportunities for niche specialization both among and within species. First and foremost, the ability to successfully process a large variety of seeds is constrained by beak size, morphology (Diaz, 1994; Johansen et al., 2014) and the related mechanics of force generation and beak movement (van der Meij and Bout, 2000). For example, larger beaks are better suited for husking larger seeds by transmitting and withstanding larger bite forces generated by the jaw muscles (Herrel et al., 2005, 2010; Soons, et al., 2010), while smaller seeds are husked more easily by smaller beaks because they allow for better seed-handling dexterity (Grant et al., 1976; Soobramoney and Perrin, 2007). As differences in morphology are typically more pronounced across species rather than within species, the link between morphology and seed preferences has mainly been studied at the species level (e.g. Abbott et al., 1975; Kear, 1962). To a much lesser extent, this has been studied at the intraspecific level, particularly in Darwin's finches in the context of temporal variation in food availability (Gibbs and Grant, 1987; Price, 1987) and in African Seedcrackers, which are known for their distinctive beak polymorphisms (Heckeberg et al., 2021). However, how kinematics of the beak, such as beak opening–closing speeds and frequencies, are related to seed preferences has received no attention so far, except for a single study on the relationship between individual seed preferences and bite force (van der Meij and Bout, 2000).

Despite the constraints imposed by beak morphology and mechanics, most bird species can still consume a large variety of edible seeds. Optimal foraging theory predicts that birds will select seeds to maximize their energy intake per unit of time (Pyke et al., 1977; Radtke, 2011). Individuals can achieve this either by preferring seeds that are more nutritious (Diaz, 1996; Marone et al., 2022), or by preferring seeds that require less time to handle (Keating et al., 1992; Titulaer et al., 2018) and/or are easier to dehusk (Soobramoney and Perrin, 2007; Van der Meij and Bout, 2000). This efficiency of seed handling in particular can be an important cause of variation in seed preferences among individuals of the same population. As a general example, juvenile birds display worse seed handling skills due to inexperience and are hence slower and less successful at feeding on more difficult-to-process seeds than adults, which causes juveniles to prefer easier-to-process seed types (Kear, 1962; Wunderle, 1991). However, seed handling times, husking success rates and seed-handling skills have been observed to vary significantly even among adult individuals (Andries et al., 2023a,b), so individual variation in seed preferences might be significant regardless of age.

Traditionally, seed preference is considered to arise in response to the need to feed efficiently, but it is possible that, in turn, seed preference also affects feeding efficiency, possibly through a positive feedback loop. As mentioned earlier, individuals can select seeds based on handling efficiency and appropriate handling skills (Kear, 1962; Titulaer et al., 2018). Better handling skills improve seed-handling time (Andries et al., 2023a,b) and skills are expected to improve with experience (e.g. Briffa and Lane, 2017). Therefore, individuals who prefer specific seeds have more experience with handling those seeds, thus might improve their handling skills faster and consequently display shorter handling times and/or higher success rates. Additionally, as developing and retaining feeding skills is energetically costly and correlated with the complexity of the neural system (Lefebvre et al., 1997; Timmermans et al., 2000), there might also be a trade-off involved where specialising in certain seed types leads to a reduced efficiency of feeding on other seed types (Peterson, 1993; Bardwell et al., 2001). However, whether seed preference affects feeding performance and whether this results in a specialization trade-off has yet to be studied.

Therefore, in this study we investigated the extent of individual variation in seed preferences within a captive population of the Canary [*Serinus canaria* (Linnaeus)] and tested whether seed preferences can influence feeding performance, associated seed-handling skills and the underlying beak kinematics during feeding on a few species-typical seed types. We consider preference for a seed type to be equivalent to having a greater proportion of that seed in the diet. As niche width is typically defined by the use of different resources (e.g. Bolnick et al., 2003), seed preference should reflect seed specialization. Under the assumption that there is a trade-off to seed specialization, we expect that individuals who specialise in a specific seed type are faster and more successful at processing that seed type, but worse at processing other seed types, compared to more generalist individuals without clear seed preferences, which are expected to perform more intermediately. In addition, if seed preferences affect feeding performance, we expect these effects to be more pronounced in adults when compared to juveniles, since they have more experience with their preferred seed types.

## RESULTS

### Individual variation in seed preference

**Fig. 1.**
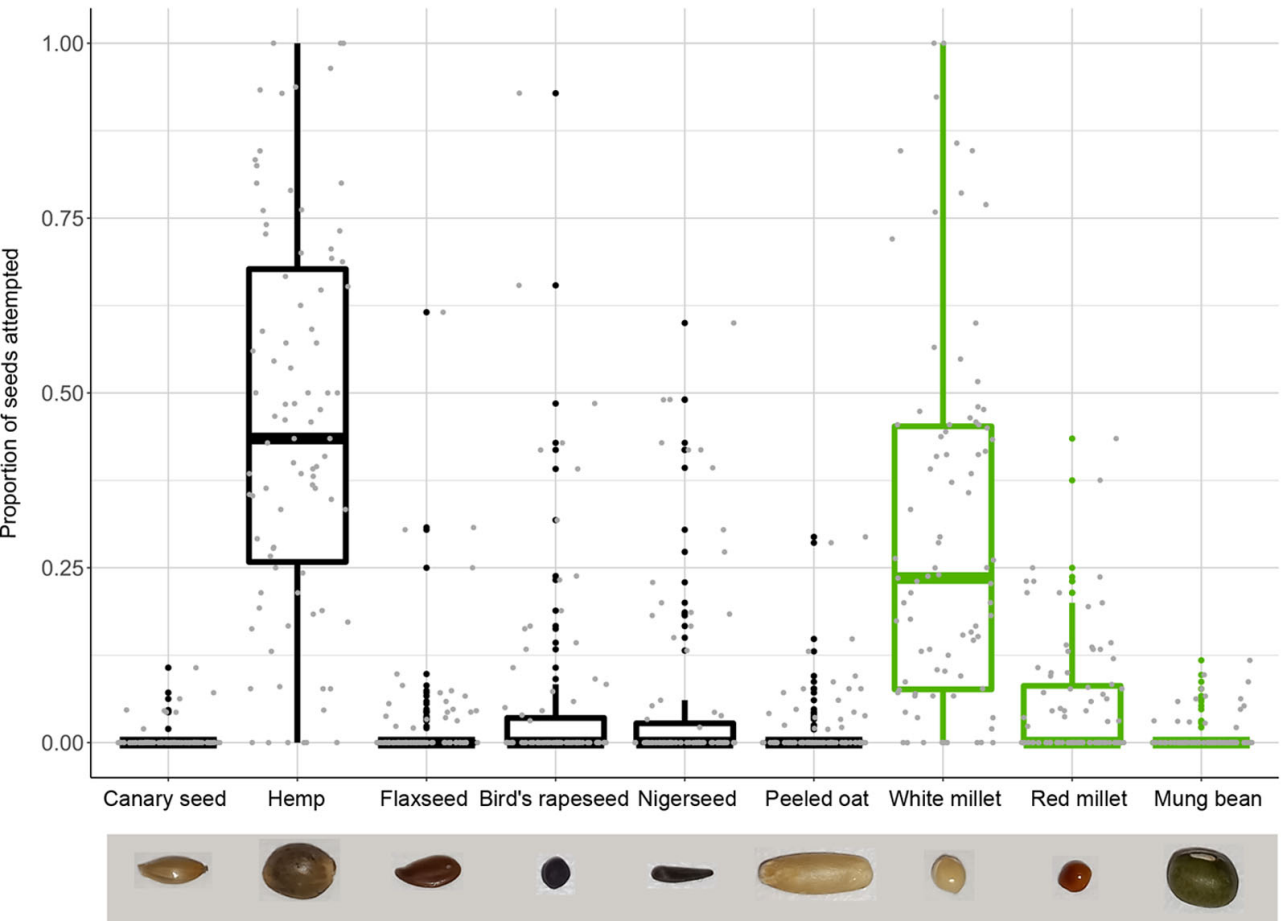
**Distribution of the proportion of seeds selected per seed type.** Selected seeds include both successful and failed seed husking attempts. Raw data points are shown as grey dots and represent individual birds (*N*=87 per seed). Novel seeds (Table 1) are coloured in green. The centre line denotes the median value (50th percentile), while the boxes contain the 25th and 75th percentiles. The black whiskers mark the 5th and 95th percentiles, and values beyond these upper and lower bounds are considered outlier values and shown as black dots. A picture of each seed is shown below their respective names.

Fig. 1 shows the distribution of seed attempts (i.e. successful and failed attempts combined) by the canaries per seed type as proportion of all seed attempts. Variation among individuals was considerable. Hemp and white millet seed showed large variation - with some individuals exclusively selecting these seeds, while others never selected them, and show a roughly equal distribution across the whole range of preference. Flaxseed, bird's rapeseed, oat, nigerseed and red millet were frequently selected by some individuals, but most did not choose them. Canary seed and mung bean were almost never selected by any individual.

**Table 1.**
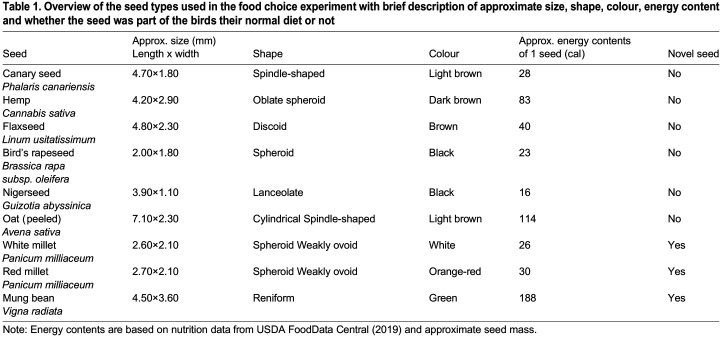
Overview of the seed types used in the food choice experiment with brief description of approximate size, shape, colour, energy content and whether the seed was part of the birds their normal diet or not

### Effects of seed preference on feeding performance, skills and beak kinematics

**Fig. 2.**
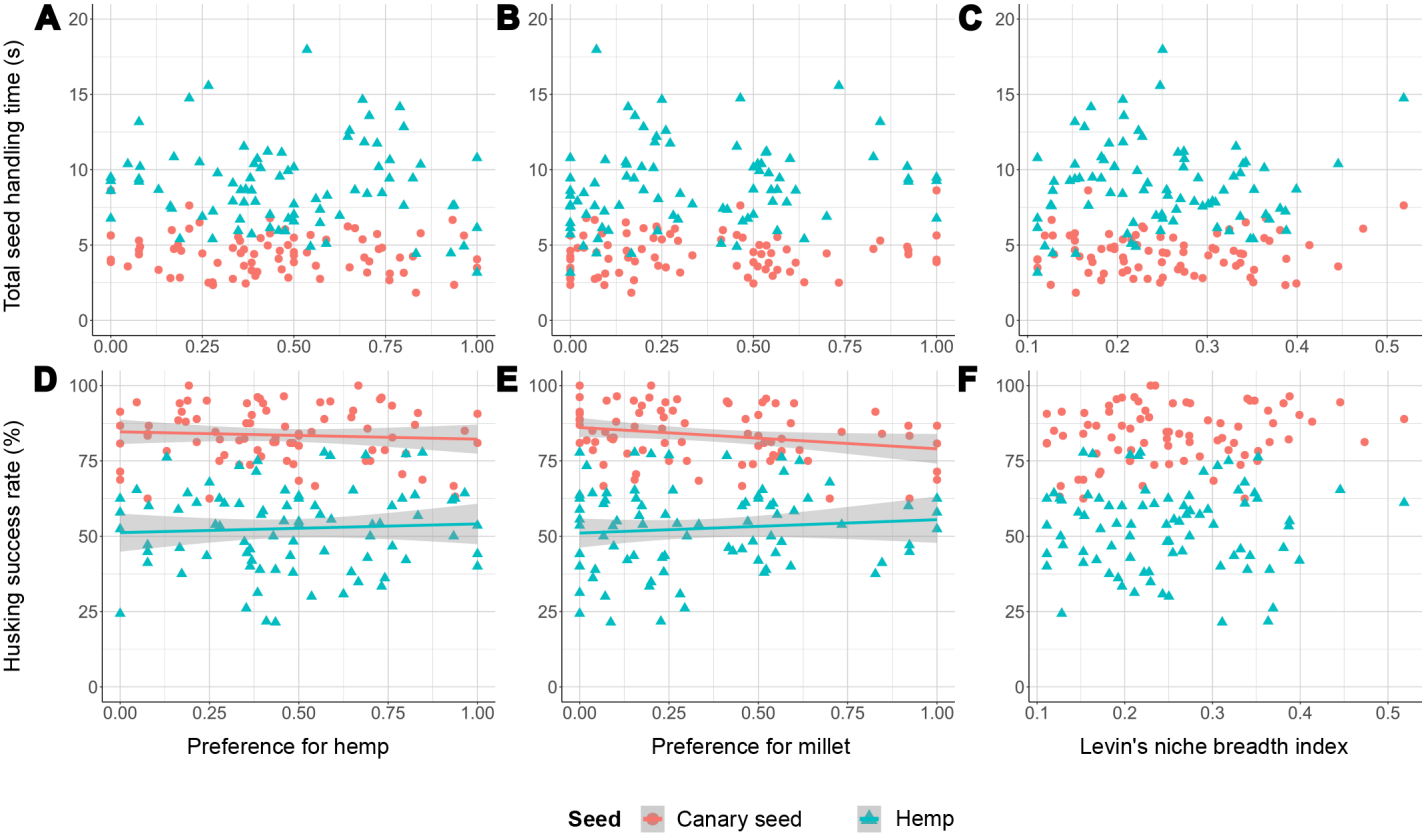
**Seed preferences in function of seed handling time (A-C) and husking success rate (D-F) during feeding on canary seed (*N*=79) and hemp seed (*N*=82).** Data points represent individual birds. Significant relationships (*P*<0.05) of the linear mixed models are represented by a regression line and its 95% confidence interval shaded in grey. Numerical and statistical results of the regression analyses can be found in Table S1.

**Fig. 3.**
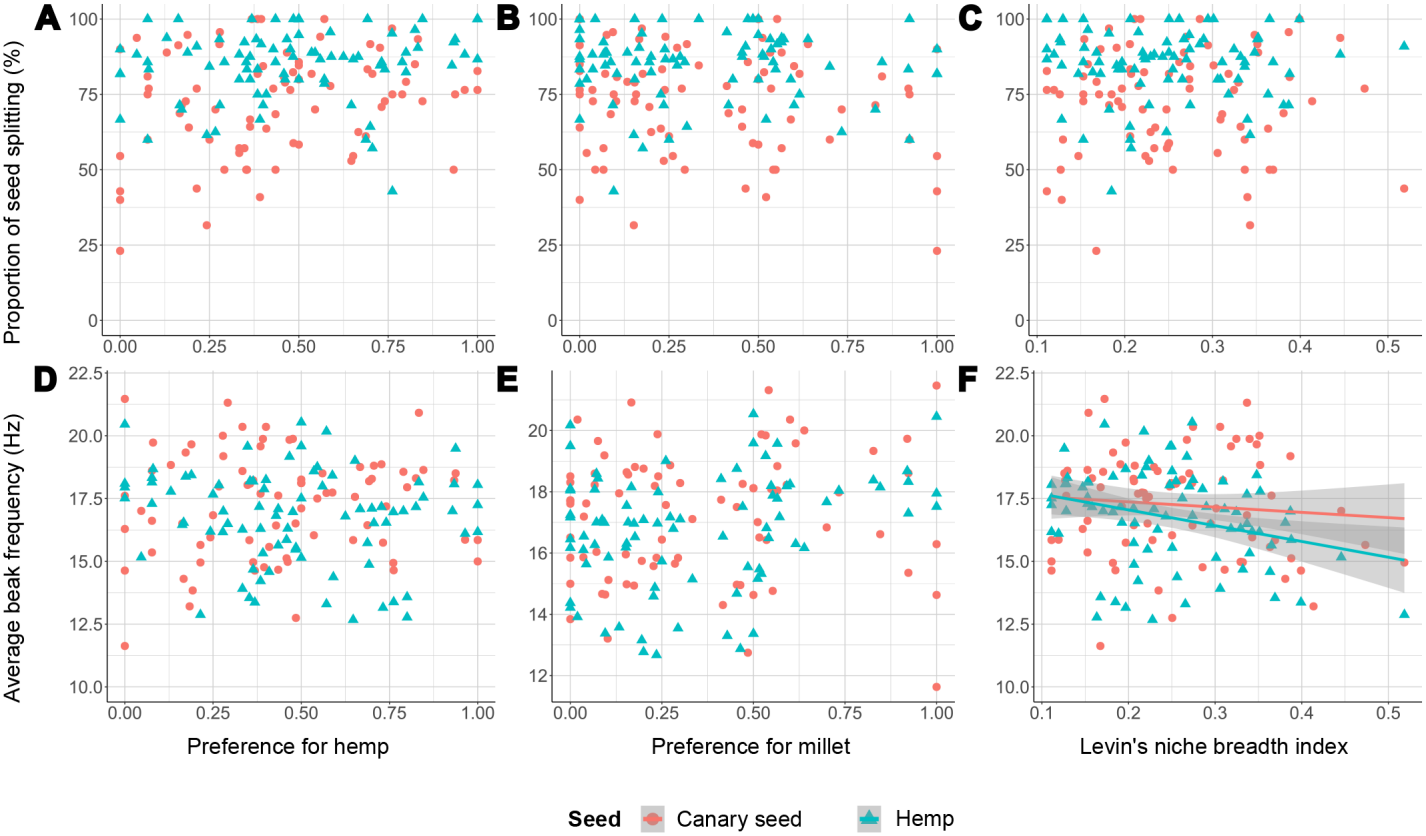
**Seed preferences in function of seed handling skills (A-C) and beak frequency (D-F) during feeding on canary seed (*N*=79) and hemp seed (*N*=82).** Data points represent individual birds. Handling skill is represented by the proportion of seed husks that are being split in half as opposed to being crushed indiscriminately (Andries et al., 2023a,b). Significant relationships (*P*<0.05) of the linear mixed models are represented by a regression line and its 95% confidence interval shaded in grey. Numerical and statistical results of the regression analyses can be found in Table S1.

Only few significant effects were observed in our linear mixed models (*P*<0.05, see Table S1). Manly's selectivity index for hemp (i.e. preference for hemp seed) had an effect on husking success rate in its interaction with the two experimental seed types [Manly(hemp)* seed_type, *P*=0.015], indicating that individuals that strongly preferred hemp seed were slightly more successful at husking hemp seeds than individuals with no preference for hemp seed, while individuals with a stronger preference for hemp seed were slightly less successful at husking canary seeds (Fig. 2D). Manly's selectivity index for millet also had an effect on husking success rate of both canary seed and hemp seed [Manly(millet), *P*=0.014], as well as its interaction with the two experimental seed types [Manly(millet)* seed_type, *P*=0.003]. This indicates that individuals with a strong preference for millet seed were less successful at husking canary seeds, but more successful at husking hemp seeds than individuals that avoided millet seeds (Fig. 2E). The degree of seed specialization, represented by Levin's niche breadth index, only had a significant effect on beak frequency in its interaction with the two experimental seed types (Levin*seed_type, *P*=0.032). Specifically, more generalist individuals opened and closed their beaks more slowly (during the act of seed positioning, see Andries et al., 2023a,b) than more specialist individuals, but only during feeding on hemp seeds (Fig. 3F). Lastly, no interactions with age were significant, hence no effects of either hemp preference, millet preference or degree of seed specialization differed between juveniles and adults. There was no overall effect of age either.

## DISCUSSION

How seed preferences in granivorous songbirds are affected by morphological and mechanical constraints (van der Meij and Bout, 2000), feeding performance (Keating et al., 1992; Titulaer et al., 2018) and seed handling skills (Wunderle, 1991) is a well-studied subject in evolutionary and behavioural biology. However, here we investigated this relationship from the inverse perspective and tested whether seed preference itself can also affect performance, skills and kinematics of seed feeding. Using a lab-based canary population, we found considerable variation in seed preferences among individuals. Particularly, preferences for hemp seed and the unfamiliar millet seed varied from individuals fully avoiding these seeds to others exclusively selecting them. However, we found no clear evidence that variation in seed preferences meaningfully affected feeding performance, skills or kinematics.

### Substantial individual variation in seed preference

Many birds species feed on a wide variety of seed types, differing in size, morphology, nutritional value and difficulty of being dehusked (van der Meij and Bout, 2000). Consequently, individuals of the same species may differ in seed preferences by prioritizing different seed characteristics. Here, we show that individual songbirds from a captive population do vary substantially in their seed preferences.

Intriguingly, individuals varied a lot in their preference for hemp seed (Fig. 1). Hemp seeds are high in calories (Table 1) and typically preferred food items in canaries (Kear, 1962). However, hemp seeds are relatively large and are therefore difficult and time-consuming to dehusk (Andries et al., 2023a,b). Thus, there could be a trade-off between energetic content and feeding effort, where individuals that prioritize feeding on fewer, but more nutritious seeds show a stronger preference for hemp seeds.

Curiously, almost all birds completely avoided canary seeds (Fig. 1). This not only contradicts earlier food choice experiments on wild-caught canaries, where canary seed was the second-most preferred seed after hemp seed (Kear, 1962), but it is also surprising from an ecological perspective. While canary seeds contain relatively few calories (Table 1), they are open-shelled seeds (unlike the other seeds provided) and thus easy to dehusk (Andries et al., 2023a,b). Thus, we would expect canary seed to be favoured by individuals that prioritize quantity over quality, such as juveniles (Wunderle, 1991). However, it is possible that among the seed types offered, individuals favoured bird's rapeseed and nigerseed instead, which might be even easier to dehusk than canary seed, despite being somewhat less nutritious (Table 1). While we do not have the data to confirm this ourselves, Kear (1962) did report that canaries are faster at dehusking bird's rapeseed than canary seed.

Perhaps most puzzling is the large variation of preference for millet seed (Fig. 1). Millet seeds are relatively low in calories (Table 1) and our birds had no prior experience with these seeds (it is not part of their normal diet). Since canaries have a tendency to avoid novel seed types (Doherty and Cowie, 1994), we would thus expect millet seeds to be largely avoided. However, some studies argue that seed size and morphology are the most important characteristics in seed selection (Diaz, 1996; Soobramoney and Perrin, 2007). Since millet seeds are round and relatively small (Table 1), bolder individuals might select them, with the expectation that they are easy to dehusk. There might be some merit to this hypothesis, as Kear (1962) reported that handling times of millet seed by wild-caught canaries are similar to handling times of canary seed. Why these individuals do not instead choose rapeseed, another small and round seed, might then be related to preferences in colour (Table 1; Slaby and Slaby, 1977; Keating, 1989).

### Seed preferences hardly affect feeding performance

Despite individuals differing substantially in their seed preferences, these preferences had no substantial effect on their feeding performance, skills or beak kinematics whilst feeding on either canary seed or hemp seed. This contradicts our hypothesis, since we expected individuals with a strong preference for a seed type to be faster or more successful at husking that seed type than individuals with weaker preference. While we did find some significant effects of both preference of hemp and millet seeds on the success rate of seed dehusking, these effects were rather small. For example, differences in success rate of 10–15% at most were found between individuals with very strong preference and no preference (Fig. 2D,E). Similarly, the frequency of beak opening and closing during seed positioning only decreased with roughly 3 Hz between the most and least specialized individuals (Fig. 3F). Considering that the total individual variation in frequency ranges from 12 to 22 Hz, this effect is also relatively minor. Hence, we consider the effects of seed preference on either feeding performance, skills or beak kinematics to be of only minor ecological relevance. If seed preference is an accurate proxy of seed specialization, this would imply that specializing in specific seed types does not substantially improve feeding efficiency. However, to confirm whether our preference measures, which are ultimately a snapshot, accurately reflect lifetime seed preferences and specialization, we should ideally have repeated experiments over extended time periods, but this was not feasible for this study.

Still, there might be mechanistic explanations for the limited effects of seed preference on feeding performance. There is a strong positive effect of seed-handling skill on feeding performance (Andries et al., 2023a,b). It is logical to assume that these skills are primarily trained through experience, but the sensorimotor feedback system for seed processing in granivorous songbirds could also be developmentally canalized and seed handling skills could therefore develop regardless of training. This, however, seems rather unlikely since canaries have been observed to learn how to dehusk seeds efficiently from their parents, at least during early life (Cadieu et al., 1995, 2008). Instead, it might be possible that the sensorimotor feedback system is robust enough that skills trained by feeding on certain seed types can easily be transferred to other seed types. This seems more plausible as individuals (of the same species) appear to position seeds between the lower and upper beak for cracking in a similar fashion, regardless of seed shape or size (Andries et al., 2023a,b; Kear, 1962). Alternatively, handling skills might still be seed-specific (e.g. differences between open- and close-shelled seeds, see Kear, 1962), but granivorous songbirds could be very fast learners and only limited experience may be required to handle new seed types efficiently enough, while effects of further experience quickly becomes negligible. Longitudinal studies to assess feeding skills, and studies where individuals are raised on restricted seed diets might shed more light on how feeding skills develop in granivorous songbirds and how different seed types can play a role in this.

### The role of social context in seed preference

It should be noted that the seed preferences we observed in this study might be affected by social context to a certain degree. Domestic canaries are group-living animals with a social hierarchy that is apparent in competition for food (Shoemaker, 1939; Parisot et al., 2004). Specifically, more dominant individuals spend more time at feeders and win more interactions with competing individuals (Tanvez et al., 2008). Hence, individuals might develop a socially enforced seed choice to reduce competition among them, where more dominant individuals can feed on the most-desired seed types, while more subordinate individuals make do with what is left. However, during our food choice experiments, individuals fed alone, so any social-hierarchy effects were probably absent. Potentially relieved from forced seed choice, individuals might instead choose to act opportunistically and select different seed types than they would usually select (e.g. birds that fed on a restricted seed diet increased their intake of different seeds when given the opportunity, see Kear, 1962). In this case, birds might not necessarily have more experience with the seeds they selected during our experiment. Our mechanistic explanations for the limited effects of seed preference on feeding efficiency are still plausible, especially while group sizes are small and food is available *ad libitum*. However, we want to highlight that the process of seed choice in granivorous songbirds is possibly even more complex and context-dependent than previously assumed. This underscores the importance of lab-based studies where social context can be controlled for future research. As such, it would be interesting to test individual seed choice in a group setting to investigate whether it differs in function of the preferences of the other group members and an individual's own dominance rank, though larger group sizes might become challenging to monitor.

### Conclusions

We found substantial variation in individual seed preferences in a population of canaries, demonstrating that different individuals prioritize different aspects of seed choice, such as seed quality, feeding effort and tendencies to explore new food items. Surprisingly, these preferences only had limited effects on feeding efficiency, implying that specializing in specific seed types does not substantially improve feeding efficiency. These results suggest that seed-handling skills, which enhance feeding efficiency, are easily transferable across various seed types, or that acquiring proficient seed-handling skills for different seed types requires minimal experience. Conducting longitudinal studies to evaluate feeding skills and raising birds on restricted-seed diets could provide further valuable insights into the development of feeding skills in granivorous songbirds. Additionally, seed choice in canaries is possibly affected by social context to a degree, adding a layer of complexity to the process of seed selection and highlighting the importance of controlled, lab-based studies.

## MATERIALS AND METHODS

### Study species

For this study we used individuals of a captive population of Fife Fancy Canaries [*Serinus canaria* (Linnaeus)] as model system. As this study is a follow-up of Andries et al. (2023a,b) where feeding performance, skill and beak kinematic variables of canaries were measured, we used the same 87 individuals (47 males and 40 females; all birds were between 3 months and 4 years old) for this study. Birds were housed in single-sex aviaries (1×2×2.3 m) at a room temperature of 19–24°C and a 12 h:12 h day:night cycle with food and water *ad libitum*. On the day of the experiment, birds were kept in individual test enclosures for at least 4 h to habituate them. Water was available *ad libitum* during this period, but birds were deprived of food to stimulate feeding during the experiment. The test enclosures were near-identical to those described by Andries et al. (2023a,b), consisting of a square glass box (50×50×50 cm) with the top covered by a metal grating, the floor covered with shell grit and containing a perch spanning the width of the enclosure for additional comfort. Different from the setup of Andries et al. (2023a,b), the test enclosure did not contain a tripod with food receptacle since the food was presented on the floor of the enclosure (see below).

### Experimental set-up

**Fig. 4.**
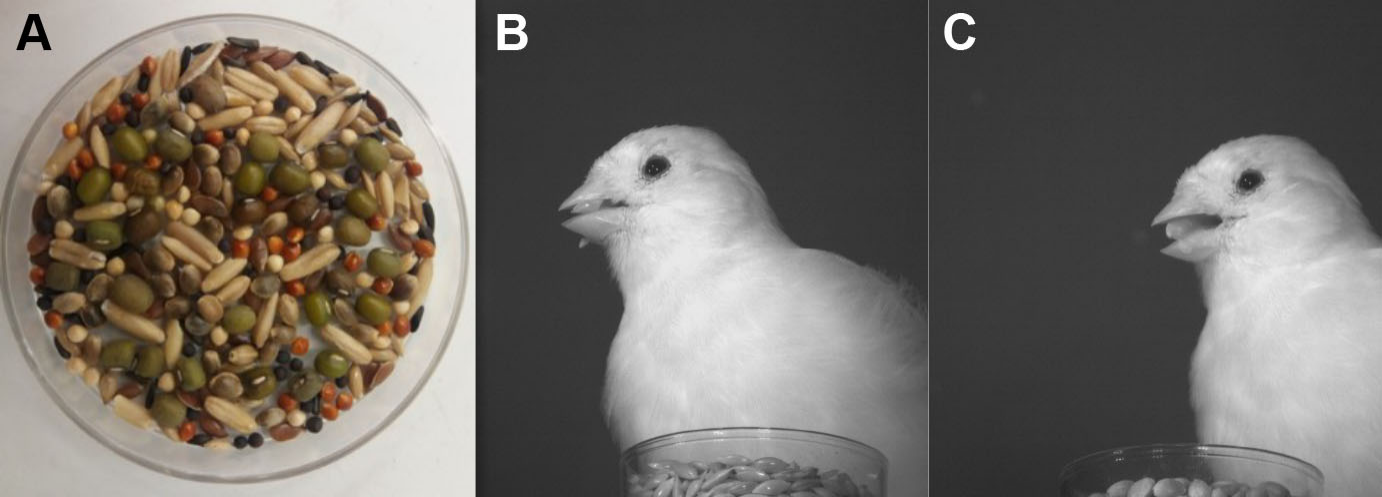
**(A) Tray with the nine different seed types used during the food-choice experiments.** (B,C) Single frame from high speed recordings of a bird feeding on canary seed (B) and hemp seed (C), used for gathering data on feeding performance, skills and beak kinematics (see Andries et al., 2023a,b).

To assess food preferences, we conducted food choice experiments. Individual birds were presented with a tray on the ground containing nine different seed types (see Table 1; Fig. 4). Seeds were specifically chosen to have a variety in size, shape, colour and a mix of familiar and novel seed types, with respect to the birds’ regular diet. The tray contained 50 seeds of each type to avoid birds running out of any one seed type, which could lead to selection bias (the maximum number of seeds of a single type eaten by one individual was 35). After presenting the tray, birds were allowed to feed for ten minutes while being filmed by a Sony RX1000M4 camera. During the recording, the bird was left alone in the room to reduce stress and avoid distractions. All food-choice experiments were conducted within a week of the experiments described in Andries et al. (2023a,b). Ethical approval for this experiment was granted by the Ethical Committee for Animal Testing of the University of Antwerp (approval number: 2021-35).

Afterwards the recordings were used to manually count how many seeds of each type were selected per individual. As we were interested in the behaviour of seed selection rather than actual seed consumption, we counted both successful and failed feeding attempts. However, considering that granivorous birds often do not readily give up a seed after a failed husking attempt (Greig-Smith, 1987), instances where a bird picked up the exact same seed after a failed attempt were not counted as separate choices. Instances where a bird picked up and dropped a seed without displaying any manipulations were not counted either. Data on feeding performance, beak kinematics and feeding skills from the same individual during feeding on two seed types (canary seed and hemp seed) were taken from Andries et al. (2023a,b) (Fig. 4).

### Preference, skill, performance and kinematic metrics

To simplify the analyses, we decided to condense our preference data into three informative metrics. Firstly, we calculated Manly's selectivity index for hemp seed, assuming no food depletion since birds never ran out of any seed type during the experiment (Chesson, 1983; Titulaer et al., 2018). This index is a metric of preference and was calculated using the following formula:
(1)

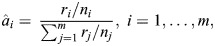
where 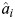 is the seed preference, *r*_*i*_ is the number of seed type *i* that was selected, and *n*_*i*_ is the total number of seed type *i* that was offered (likewise for *r*_*j*_, *n*_*j*_ and seed type *j*). This index is a value between 0 and 1 where 0 indicates that seed *i* was not selected, while 1 indicates that the individual only selected seed *i*.

We chose hemp seed as we observed a large variation in preference among individuals (see Results). Furthermore, we have additional data on feeding performance, kinematics and skill for feeding on hemp seed (Andries et al., 2023a,b), which facilitates the interpretation of any observed relationships. Secondly, we calculated Manly's selectivity index for millet seed. Similarly, we observed large variation in preference for this seed. As this is also a seed type the birds had no previous experience with, it can be regarded as a proxy for curiosity or innovation. We combined both white and red millet in this metric as both seed types are nearly identical in everything except colour (see Table 1). Lastly, we calculated Levin's niche breadth index (Feinsinger et al., 1981), which is a metric for how generalist or specialist an individual is, regardless of which seed types it would specialise in, and was calculated using this formula:
(2)

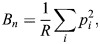
where *B*_*n*_ is Levin's niche breadth index for an individual, R is the number of different seed types available, and *p*_*i*_ is the proportion of seed type *i* selected by the individual. This index is a value between 0 and 1 where the higher the value, the more generalist the individual is.

Feeding performance, kinematics and skill data were taken from Andries et al. (2023a,b). In order to limit the number of statistical tests, we only used the ecologically most relevant variables (Andries et al., 2023a,b): two feeding performance metrics, namely average seed handling time and husking success rate, the proportion of seed husks split as opposed to crushed as a representation of skill and the average frequency of beak opening and closing during seed positioning as kinematic metric (see also Andries et al., 2023a,b).

### Statistical analyses

Since we aim to investigate whether food preference can affect feeding performance, we constructed linear mixed models with seed handling time, husking success rate, skill and frequency as response variables and seed preference metrics as predictor variables {Manly's selectivity index for hemp seed [Manly(hemp)], Manly's selectivity index for millet seed [Manly(millet)] and Levin's niche breadth index (Levin)}. As we have performance data of feeding on two seeds (canary seed and hemp seed) and we expect effects to differ between these seed types for a given preference metric, we added seed type as a predictor variable, including interactions with the three seed-preference variables. Consequently, we had to add individual bird identity as a random effect since most individuals fed on both seed types. We also wanted to assess whether patterns are different between juveniles and adults, so we added age as a predictor variable, again including interactions with the three seed preference variables. We considered age as a categorical variable with two levels: juvenile and adult, based on the average age of maturity of 10–12 months (Austad, 2011). Since none of our individuals were close to this boundary age, we could clearly categorize our individuals as either juvenile or adult. In summary, we ran four identical mixed linear models, one for each response variable: total seed handling time, husking success rate, seed handling skill and beak frequency:

∼ Manly(hemp)+Manly(millet)+Levin+seed_type+age+Manly(hemp)× seed_type+Manly(millet)×seed_type+Levin×seed_type+Manly(hemp)×age+ Manly(millet)*age+Levin×age.

None of the main predictor variables showed strong collinearity (highest VIF=2.43). Residuals of all four models were checked for normality and heteroscedasticity using QQ-plots and residual plots respectively. All statistical analyses were done in R (version 4.2.1.). The package ‘MicroNiche’ was used to calculate Levin's niche breadth index (Feinsinger et al., 1981) and packages ‘lme4’ (Bates et al., 2015) and ‘lmerTest’ (Kuznetsova et al., 2017) were used for the linear mixed models.

## Supplementary Material

10.1242/biolopen.060353_sup1 Supplementary information
