# Supplementary material for: Seed preference is only weakly linked to seed-type-specific feeding performance in a songbird
Source: Biol Open. 2024 Apr 11;13(4):bio060353. doi: 10.1242/bio.060353 (PMC11033522; doi:10.1242/bio.060353)
Supplement: Supplementary information [file biolopen-13-060353-s1.pdf]

Table S1. Summary statistics of the independent variables per regression model. Significant p-values are indicated in bold.

Variable	Slope estimate	Standard error	t-value	p-value
**Handling time (s)**				
Manly (hemp)	0.152	1.820	0.083	0.934
Manly (millet)	0.655	1.820	0.444	0.658
Levin	2.440	4.070	0.600	0.114
Seed	3.227	2.120	1.522	0.132
Age	1.868	2.345	0.797	0.428
Manly (hemp)*seed	1.156	1.898	O.609	0.544
Manly (millet)*seed	1.801	1.592	1.131	0.262
Levin*seed	0.535	4.394	0.122	0.903
Manly (hemp)*age	-0.771	2.098	-0.367	0.714
Manly (millet)*age	-0.430	1.801	-0.239	0.812
Levin*age	-3.306	4.859	-0.680	0.498
**Success rate (%)**				
Manly (hemp)	-14.116	9.029	-1.563	0.120
Manly (millet)	-18.126	7.313	-2.479	**0.014**
Levin	4.160	20.248	0.205	0.838
Seed	-53.829	11.214	-4.800	**$3.82*10^{-6}$**
Age	-11.523	11.328	-1.017	0.311
Manly (hemp)*seed	24.661	10.034	2.458	**0.015**
Manly (millet)*seed	25.703	8.428	3.050	**0.003**
Levin*seed	8.993	23.214	0.387	0.699
Manly (hemp)*age	3.166	10.136	0.312	0.755
Manly (millet)*age	4.600	8.692	0.529	0.597
Levin*age	3.325	23.519	0.141	0.888
**Seed splitting proportion (%)**				
Manly (hemp)	6.972	11.662	0.598	0.551
Manly (millet)	-10.376	9.454	-1.098	0.275
Levin	19.503	26.055	0.749	0.456
Seed	13.684	13.411	1.020	0.311
Age	-19.554	15.092	-1.296	0.199
Manly (hemp)*seed	-4.336	12.005	-0.361	0.719
Manly (millet)*seed	4.785	10.068	0.475	0.636
Levin*seed	-7.668	27.793	-0.276	0.783
Manly (hemp)*age	15.751	13.501	1.167	0.247
Manly (millet)*age	13.258	11.591	1.144	0.256
Levin*age	0.069	31.266	0.002	0.998

Variable	Slope estimate	Standard error	t-value	p-value
**Frequency (Hz)**				
Manly (hemp)	-0.148	1.598	-0.092	0.927
Manly (millet)	-0.507	1.301	-0.390	0.698
Levin	-1.515	3.539	-0.428	0.670
Seed	1.785	1.512	1.181	0.242
Age	-2.842	2.185	-1.300	0.198
Manly (hemp)*seed	-1.668	1.354	-1.232	0.222
Manly (millet)*seed	0.384	1.132	0.339	0.736
Levin*seed	-6.865	3.141	-2.186	**0.032**
Manly (hemp)*age	1.474	1.954	0.754	0.453
Manly (millet)*age	2.159	1.682	1.284	0.204
Levin*age	1.515	4.507	0.336	0.738

Seed preference is only weakly linked to Seed-type-specific feeding performance In a songbird

Biology Open • Supplementary information
